# Japan’s emerging role in bridging immunisation gap and supporting global health

**DOI:** 10.1136/bmjgh-2025-020316

**Published:** 2025-09-14

**Authors:** Aomi Katagiri, Yuka Iijima, Yoshiki Tanaka, Hikari Ohyama, Takuya Nakazawa, Hayase Hakariya

**Affiliations:** 1 Institute of Science Tokyo, Tokyo, Japan; 2 NGO Reaching Zero-Dose Children, Tokyo, Japan; 3 Department of International Health, Johns Hopkins Bloomberg School of Public Health, Baltimore, Maryland, USA; 4 Graduate School of Infomatics, Kyoto University, Kyoto, Japan; 5 Institute for Pharmaceutical and Social Health Sciences, Ise, Japan; 6 Yokohama University of Pharmacy, Yokohama, Kanagawa, Japan; 7 Graduate School of Interdisciplinary Science and Engineering in Health Systems, Okayama University, Okayama, Japan; 8 Eberhard Karls University Tubingen, Interfaculty Institute of Biochemistry, Tübingen, BW, Germany

**Keywords:** Immunisation, Vaccines, Universal Health Care, Global Health, Health policies and all other topics

**Summary box**

- The abrupt US suspension of funding to the US Agency for International Development and Gavi in 2025 threatens routine immunisation and child health programmes in low- and middle-income countries (LMICs), particularly in the Global South.
- Historical data show that US global health assistance significantly reduced under-five mortality, underscoring the potential adverse consequences of the funding freeze.
- Other major donor countries, including the UK, France and Germany, are also scaling back aid, citing domestic priorities such as defence and fiscal constraints.
- Japan, despite rising nationalism and domestic scepticism towards foreign aid, has reaffirmed its global health leadership through renewed commitments, including a major contribution to Gavi’s African vaccine manufacturing accelerator.
- Japan’s longstanding expertise in maternal and child health, its universal health coverage (UHC) advocacy and recent initiatives such as the UHC knowledge hub position it to help fill the funding gap and support sustainable health systems in LMICs even amid the contemporary unpredictable global landscape.

## Introduction

The US has long supported global immunisation through bilateral funding via the US Agency for International Development (USAID) and the Centres for Disease Control and Prevention (CDC) and multilateral funding to Gavi, the Vaccine Alliance (Gavi). However, these programmes face a crisis after the abrupt funding suspension in 2025.

## Implications of the funding freeze on global childhood immunisation programmes

After the USAID funding halt was initially announced as a 90-day suspension on 20 January 2025,^1 2^ a follow-up announcement on 10 March 2025 confirmed that 83% of USAID programmes had been terminated, and the remaining programmes would be transferred to the State Department.^3^ As of the end of May 2025, the USA has indicated that its review of foreign assistance is still ongoing.^4^ In the fiscal year 2024, USAID’s budget totalled US$32.5 billion, with US$8.9 billion for health,^5^ including US$290 million for vaccine development and immunisation, which are critical life-saving interventions for children.^5^ Countries receiving above-average USAID support from 2000 to 2016 saw 29 fewer under-five deaths per 1000 live births compared with similar countries without such support,^6^ highlighting the potential consequences of this sudden cessation.

Subsequently, the USA announced that it would terminate funding for Gavi on 24 March 2025. Since its 2000 establishment, the USA has contributed US$3.95 billion (excluding contribution to COVID-19 Vaccines Global Access (COVAX)), accounting for 13% of Gavi’s budget.^7^ Gavi has facilitated immunisation for over 1 billion children, saving more than 17 million lives between 2000 and 2020.^8^ To accelerate its successful impact, Gavi has worked on its replenishment for Gavi 6.0 (2026–2030) and received a pledge from the previous US government in 2024.^9^

These US funding withdrawals threaten routine immunisation in low- and middle-income countries (LMICs), particularly in the Global South, which relies on external funding, and among vulnerable populations, including children and those in remote healthcare access.^10^ To mitigate immediate impacts, donor governments and organisations should consider emergency funding mechanisms. However, donor countries such as the UK, France and Germany are also scaling back aid spending.^5^ The UK stated that this shift is intended to provide better value for taxpayers' money and mentioned plans to increase defence spending,^11^ a trend that is also observed in other European countries.^12^

Herein, we discuss how Japan, another donor country, could step in to support global health efforts amid rising nationalism across donor countries.

## Bridging the funding gap: Japan’s strategic response and future outlook

Nationalistic sentiments have also gained traction among the Japanese population. In 2024, 18.4% of citizens advocated reducing the foreign aid budget, the highest level in the past decade.^13^ Similar to trends in other donor countries, this shift stems from a growing perception of financial burden among citizens. Nevertheless, Japan remains the world’s third-largest donor following the USA and Germany,^14^ with Official Development Assistance (ODA) totalling approximately US$19.6 billion in 2023, a 12% increase from the previous year.^15^

Historically, Japan has played a leading role in global health. Since the 1990s, it has been among the top ODA contributors, sharing its postwar experience of dramatically improving health standards and longevity through the introduction of universal health insurance in 1961. This achievement laid the foundation for Japan’s global leadership in promoting universal health coverage (UHC). Japan notably advocated for UHC at the G8 summits in Kyushu-Okinawa (2000) and in Hokkaido Toyako (2008), with a particular focus on maternal and child health in LMICs.^15^ Japan has since consistently emphasised UHC in successive G7 summits.^15^

Japan has consistently been a major contributor to the WHO, ranking 10th overall and third in annual membership fees.^16 17^ In addition to its core contributions, Japan has taken the leadership role by launching and advancing key strategic initiatives in emergency responses, such as during the COVID-19 pandemic, natural disasters and refugee crises.^17^ Remarkably, during the COVID-19 pandemic, Japan contributed an outstanding US$1.5 billion to COVAX between 2020 and 2023, more than five times its total contributions to Gavi since 2011.^9^ This extraordinary support enabled the delivery of approximately 44 million COVID-19 vaccine doses to LMICs,^18^ underscoring Japan’s leadership in emergency response.

Despite a growing nationalist discourse and waning domestic support for international cooperation, Japan announced a US$30 million contribution to the African Vaccine Manufacturing Accelerator in June 2024,^19^ a Gavi-led initiative to accelerate sustainable vaccine production in Africa. This demonstrates Japan’s consistent commitment towards global health. As expected, the Ministry of Foreign Affairs of Japan (MOFA) reaffirmed its engagement in global health in response to the US’s announcement of the USAID freeze, emphasising continued communication with international partners as of 28 February 2025.^20^ In March 2025, the MOFA released the 2024 Development Cooperation White Paper, underscoring Japan’s dedication to maternal and child health, including sexual and reproductive health services,^19^ sectors most vulnerable to the fallout of the US funding suspension.

As Japan reasserts its leadership in UHC and maternal and child healthcare, it is expected to take a pivotal role in addressing the urgent funding gap due to the US’s funding freeze. To avert predictable and preventable loss of life, immediate, medium and long-term support are essential. In the short term, it is crucial to rapidly identify impacted areas and projects, allocate aid to frontline non-governmental organisations and ensure the timely distribution of essential supplies, including vaccines and medicines, but also food for those facing starvation.^19^ In the medium term, particularly in the field of maternal and child health, Japan is expected to leverage its expertise and experience in promoting the maternal and child health handbook to strengthen the continuum of care for women and children—a model implemented in countries such as Indonesia and Palestine.^19^ In the long term, Japan can support LMICs in building self-reliant and sustainable health systems through domestic healthcare financing and investment in human resources to build resilience against future shocks. In this context, Japan’s launch of the UHC knowledge hub in 2025 is a timely initiative. The hub collaborates with organisations such as the WHO and the World Bank to establish a global centre for data compilation and capacity building aimed at advancing UHC in LMICs. It plans to provide training on health financing for officials from LMIC Ministries of Health and Finance, while also sharing Japan’s experience in sustaining UHC.^21^

## Conclusion

In conclusion, amid the crisis of the US funding suspension, a widening demand gap threatens millions of marginalised lives. Japan is thus expected to take on a greater leadership role in bridging this shortfall and safeguarding the progress made in global health and health equity.

## Data Availability

All data relevant to the study are included in the article.
